# Silencing of Chemosensory Protein Gene NlugCSP8 by RNAi Induces Declining Behavioral Responses of *Nilaparvata lugens*

**DOI:** 10.3389/fphys.2018.00379

**Published:** 2018-04-12

**Authors:** Muhammad I. Waris, Aneela Younas, Muhammad T. ul Qamar, Liu Hao, Asif Ameen, Saqib Ali, Hazem Elewa Abdelnabby, Fang-Fang Zeng, Man-Qun Wang

**Affiliations:** ^1^Hubei Insect Resources Utilization and Sustainable Pest Management Key Laboratory, College of Plant Science and Technology, Huazhong Agricultural University, Wuhan, China; ^2^College of Informatics, Huazhong Agricultural University, Wuhan, China; ^3^College of Agronomy and Biotechnology, China Agricultural University, Beijing, China; ^4^Department of Plant Protection, Faculty of Agriculture, Benha University, Banha, Egypt

**Keywords:** *Nilaparvata lugens*, chemosensory protein, expression patterns, competitive binding assay, behavioral trial, RNA interference, molecular docking

## Abstract

Chemosensory proteins (CSPs) play imperative functions in chemical and biochemical signaling of insects, as they distinguish and transfer ecological chemical indications to a sensory system in order to initiate behavioral responses. The brown planthopper (BPH), *Nilaparvata lugens* Stål (Hemiptera: Delphacidae), has emerged as the most destructive pest, causing serious damage to rice in extensive areas throughout Asia. Biotic characteristics like monophagy, dual wing forms, and annual long-distance migration imply a critical role of chemoreception in *N. lugens*. In this study, we cloned the full-length CSP8 gene from *N. lugens*. Protein sequence analysis indicated that NlugCSP8 shared high sequence resemblance with the CSPs of other insect family members and had the typical four-cysteine signature. Analysis of gene expression indicated that NlugCSP8 mRNA was specifically expressed in the wings of mated 3-day brachypterous females with a 175-fold difference compare to unmated 3-day brachypterous females. The NlugCSP8 mRNA was also highly expressed in the abdomen of unmated 5-day brachypterous males and correlated to the age, gender, adult wing form, and mating status. A competitive ligand-binding assay demonstrated that ligands with long chain carbon atoms, nerolidol, hexanal, and trans-2-hexenal were able to bind to NlugCSP8 in declining order of affinity. By using bioinformatics techniques, three-dimensional protein structure modeling and molecular docking, the binding sites of NlugCSP8 to the volatiles which had high binding affinity were predicted. In addition, behavioral experiments using the compounds displaying the high binding affinity for the NlugCSP8, revealed four compounds able to elicit significant behavioral responses from *N. lugens*. The *in vivo* functions of NlugCSP8 were further confirmed through the testing of RNAi and post-RNAi behavioral experiments. The results revealed that reduction in NlugCSP8 transcript abundance caused a decrease in behavioral response to representative attractants. An enhanced understanding of the NlugCSP8 is expected to contribute in the improvement of more effective and eco-friendly control strategies of BPH.

## Introduction

A considerable amount of literature has been published on insect olfactory systems. These studies recognized that olfactory systems are particularly sensitive and complex (Forêt et al., 2007; Yoshizawa et al., 2011; Gu et al., 2012; Sun L. et al., 2016). The olfactory system has immense importance in the insects because it can detect and identify a variety of chemicals from the environment (Li et al., 2017a). Investigational studies on its functions have elucidated some of the molecular components and pathways that insects utilize in identifying conspecifics, detect enemies, find mates, locate oviposition site, and to avoid natural enemies (Field et al., 2000; Bruyne and Baker, 2008; Qiao et al., 2013; Li et al., 2017b). The high specificity and sensitivity of the insect olfactory system mostly rely on the interaction between semiochemicals and different types of protein expressed in the olfactory sensilla of insects, such as sensory neuron membrane proteins (SNMPs), membrane-bound olfactory receptors (ORs) and two types of carrier protein: chemosensory proteins (CSPs) and the odorant binding proteins (OBPs) (Pelosi et al., 2006, 2017; Leal, 2013; He and He, 2014; He et al., 2014). Chemosensory proteins encompass a family of acidic, low-molecular-mass and soluble proteins in the lymph of insect olfactory receptors and probably play significant roles in insect chemoreception, such as differentiating, binding, and transporting hydrophobic chemicals from the surroundings to olfactory sensilla (Kaissling, 2001; Pelosi et al., 2005; Gong et al., 2007; Jin et al., 2017). CSPs were originally identified in the antennae of *Drosophila melanogaster* by McKenna et al. (1994). CSPs are around 100–120 residues long and present a conservative model of four cysteines forming two independent loops (Angeli et al., 1999; He et al., 2017). CSPs also have α-helical segments but accumulated in a folding different from that of insect OBPs (Jansen et al., 2007; Northey et al., 2016). Through the expressed sequence tag (EST) and transcriptome databases in addition with the development of genome comprehensive surveys, more and more CSP families and their biochemical functionality/expressions have been described in many insect species (Zhou et al., 2006). Various CSPs are known to be ubiquitously expressed in insects and shown to be interrelated with larval development, detection of carbon dioxide, and regeneration of tissues (Pelosi et al., 2006; Li et al., 2016; Iovinella et al., 2017). However, data has revealed that CSPs or CSP-like genes are expressed not only in the antennae, the main olfactory organ (Zhang et al., 2009), but also in the wings (Zhou et al., 2008), legs (Picimbon et al., 2001), pheromone glands (Dani et al., 2010), proboscis (Liu et al., 2014), as well as in all other components of insect body (Gong et al., 2007), and involved in odor recognition (Sánchez-Gracia et al., 2009). This comprehensive and varied expression pattern proposes that CSPs may play several functions, beyond chemosensation (Tegoni et al., 2004). CSPs highly enriched in antennae have proposed chemosensory functions in Lepidoptera (Qiao et al., 2013). Antennae-enriched CSP1 from *Microplitis mediator* play important functions in chemoreception and used as a potential target to regulate the olfactory behavior in *M. mediator* (Peng Y. et al., 2017). Other CSPs highly expressed in antennae have been concerned with serving functions in the behavioral phase change in *Locusta migratoria* (Guo et al., 2011). In *Spodoptera exigua*, SexiCSP3 has been associated with egg hatching and ovipositions (Gong et al., 2012), while PameCSP10 in *Periplaneta americana* appears to be the main extracellular matrix protein during limb regeneration (Kitabayashi et al., 1998). The chemosensory protein, Si-CSP1 involved in regulating the necrophoric behavior of workers in *Solenopsis invicta* (Qiu and Cheng, 2017). Numerous studies designated that CSPs may be involved in immune response, circadian cycles or developmental process (Oduol et al., 2000; McDonald and Rosbash, 2001; Sabatier et al., 2003). CSPs are, therefore, expected to perform many miscellaneous tasks from behavior to several physiological and biological processes (Pelosi et al., 2017). Ligands from different sources, such as plant volatiles (Fujii et al., 2010), cuticular lipids (González et al., 2009), cuticular hydrocarbon (Ozaki et al., 2005), and brood pheromones (Briand et al., 2002), are usually used in the fluorescence binding assays to characterize the binding affinity between CSPs and various odorants. Multiple functions proposed or documented that CSPs have the capability to bind and interact with small molecules, from nutrients to semiochemicals, toxic compounds or hormones (Pelosi et al., 2017). These extraordinarily complex binding functionality and expression profiles proposed that CSPs might play an important role in the insect chemosensory systems, while their exact physiological functions and mechanisms still remains unclear (Sánchez-Gracia et al., 2009).

The brown planthopper (BPH), *Nilaparvata lugens* (Stål) (Hemiptera: Delphacidae), is a major insect pest of rice in extensive area throughout Asia and could cause enormous economic losses (Dong et al., 2011; Bottrell and Schoenly, 2012; Peng L. et al., 2017). BPH is a monophagous herbivore that mainly feeds on cultivated rice and its associated wild rice, and therefore the strategies being used to find rice plants would be vital in BPH (Sogawa et al., 1982). In rice plants, BPH decreases the photosynthetic rate, chlorophyll content, nitrogen concentrations of stem and leaf, and organic dry weight, thereby intensively decreasing yield (Ye et al., 2017). In the adult stage, BPH shows two wing forms, short (brachypterous) and long (macropterous) ones. The long wing adults exhibit the capability to migrate across long distances, while the short wing adults expound strong reproductive abilities (Bottrell and Schoenly, 2012; Cheng et al., 2013). These biotic characteristics imply the critical role of chemoreception in BPH. However so far, limited olfactory-interrelated proteins have been categorized in *N. lugens*. Total of 10 genes encoding OBPs (*NlugOBP1*-*10*) and 11 genes encoding CSPs (*NlugCSP1-11*) are predicted from the genome in previous studies (Xu et al., 2009; He et al., 2011; Yang et al., 2014; Zhou et al., 2014). Of these predicted genes, only one CSP gene (*NlugCSP7*) has been cloned from the antennae of *N. lugens* and subsequently identified as volatile organic compound binding capabilities (Yang et al., 2014). However, previous ligand-binding analysis of NlugCSP7 revealed that it may possess physiological functions other than the chemosensation (Yang et al., 2014). The functions of other chemosensory related proteins are still unknown in *N. lugens*. To date, very little attention has been paid to the functions of *N. lugens* chemosensory related proteins. Previous studies also demonstrated that NlugCSP8 may play roles in perception of rice plant volatiles after the *N. lugens* dispersion (Yang et al., 2014). To confirm their specific functional roles, we conducted a more thorough study of NlugCSP8 expression and functionality. The main objective of this paper is to recognize the functions of NlugCSP8 during development. We performed qRT-PCR to monitor the expression of NlugCSP8 during different development stages of unmated and mated adults in terms of wing forms, tissues, and genders. Binding properties of NlugCSP8 were also tested using a number of ligands in fluorescence binding assay. In addition, molecular docking analyses followed by targeted gene silencing using RNAi combined with behavior bioassay were conducted.

## Materials and methods

### Insect rearing and tissue collection

Successive generations of BPH were reared on susceptible rice variety Taichung Native 1 (TN1) in a climatic chamber under constant conditions of 26 ± 2°C, 75 ± 5% relative humidity and 16-h light: 8-h dark photoperiod. For the expression pattern analysis, unmated and mated 3- and 5-days short/long wing adults of both sexes were collected. To obtain mated male and female adults, newly emerged BPH males and females were paired in glass tubes and allowed to mate. Before sample collection, the age of adults was checked and confirmed according to previous literature (Wipfler et al., 2016). The tissues were dissected from antennae, head (without olfactory appendages), abdomen, legs, and wings of unmated and mated short and long wing adults of both sexes and collected for qRT-PCR. All samples with three replicates (50 individuals per replication) were kept at −80°C and further arranged according to age, mating status, and sex.

### RNA extraction and cDNA synthesis

Total RNA was extracted from individual samples by using the TRIzol reagent (Invitrogen, CA). Then quality and quantities were examined by using 1.0% agarose gel electrophoresis and ultraviolet spectrophotometer (Eppendorf Bio Photometer Plus, Germany). The first-strand cDNA for RT-PCR and qRT-PCR were synthesized from 1 μg of total RNA using MBI RecertAid First Strand cDNA kit (MBI Fermentas, Glen Burnie, MD, USA) and PrimerScript RT Reagent kits with gDNA Eraser (Perfect Real Time; Takara) respectively, according to manufacturer's instructions. The synthesized cDNA was stored at −20°C for future use.

### NlugCSP8 sequence analysis

NlugCSP8 was identified with a complete coding sequence from our previous cDNA library (Zhou et al., 2014). The open reading frame (ORF) was recognized using the ORF finder software (http://ncbi.nlm.nih.gov/gorf/gorf.html). The molecular weight was calculated using the SWISS-PROT (ExPASy server) program “Compute pI/Mw.” The signal peptides were predicted using SignalP V3.0 (http://www.cbs.dtu.dk/services/SignalP/). NlugCSP8 similarity search to identify homologous genes from other insect species were performed using the NCBI-BLAST (http://blast.ncbi.nlm.nih.gov/) and sequences were further aligned by using ClustalX 1.83 and GeneDoc 2.7 computer programs (Thompson et al., 1997). Multiple sequence alignment has been performed and the evolutionary tree was constructed using the neighbor-joining method with MEGA 6.0 (Tamura et al., 2013).

### Quantitative RT-PCR

A quantitative RT-PCR (qRT-PCR) was used to study the spatiotemporal expression profiles of NlugCSP8 in mRNA level in unmated and mated, 3 and 5 days old, short and long wing adults of both sexes. We generated cDNA from the selected tissues of the short and long wings of both *N. lugens* sexes in different mating stages and age groups. β-actin (GenBank accession number: EU179846) was used as an internal control (Liu et al., 2008). Primer sequences were designed using the Primer 5.0 program (Premier Biosoft International, Palo Alto, CA, USA). A 10-fold dilution series was used to construct a standard curve in order to determine the qRT-PCR efficiencies and to quantifying the amount of target mRNA. In all experiments, all primers achieved amplification efficiencies of 95–100%. The qRT-PCR samples contained 10 μl of 2 × Syber Green PCR Master Mix, 0.5 μl of each primer (10 μM), 1 μl of cDNA and 8 μl sterilized ultrapure water. Thermal cycling was performed using an initial denaturation step at 95°C for 3 min, followed by 40 cycles of 95°C for 10 s and 55°C for 30 s. The qRT-PCR was performed in triplicate using three biological samples and the relative Ct-values were quantified using the 2^−ΔΔCT^ method (Livak and Schmittgen, 2001; Li et al., 2014).

### NlugCSP8 expression vector system construction

For expression of NlugCSP8 (NlugCSP8; accession no. ACJ64054.1), the sequence encoding NlugCSP8 was amplified by PCR with a forward primer containing an *EcoRI*-restriction site and a reverse primer containing an *XhoI*-restriction site (Table S1). The PCR product was ligated into a pMD-18T vector and sequenced. The pMD-18T plasmid containing target sequence flanked by the two restriction sites was digested with *EcoRI* and *XhoI* restriction enzymes and ligated into the expression vector pET-30a, which was earlier linearized with the same restriction enzymes. The obtained plasmids were sequenced and shown to encode the mature protein.

### Expression and purification of recombinant NlugCSP8

The recombinant pET-30a/CSP8 expression plasmid was transformed into *Escherichia coli* BL21 (DE3) competent cells. After DNA sequencing, a single positive clone was grown in 10 mL Luria-Bertani (LB) medium containing kanamycin (50 μg/mL) with shaking overnight at 220 rpm and 37°C. The culture was diluted to 2 L LB medium (supplemented with 50 μg/mL kanamycin) and grown at 37°C with shaking at 220 rpm until the culture reached the optical density value of ~0.6–0.7 at 600 nm. The recombinant protein expression was induced by the addition of 2 mM IPTG (Isopropyl β-D-1-thiogalactopyranoside), followed by culturing for 4 h at 37°C. The bacterial cells were harvested by centrifugation (10,000 rpm, 10 min) and sonicated. The expressed protein presented in the supernatant as a soluble form. Then, NlugCSP8 purification was performed using a Ni-ion affinity chromatography column (GE Healthcare, Uppsala, Sweden). His-tag was removed from the recombinant protein with the addition of recombinant bovine enterokinase (EK) in the eluted fractions of protein, followed by 16 h incubation at 25°C. After running the digested protein back through the Ni-ion affinity chromatography column, the tag-free protein was obtained in the flow through fraction. Protein expression and purification steps were assessed by 15% SDS-PAGE (sodium dodecyl sulfate-polyacrylamide gel electrophoresis). Finally, purified protein was dialyzed in Tris buffer (pH 5.0) and (pH 7.4). The concentration of purified protein was determined prior to perform ligand-binding specificities of NlugCSP8 with 25 selected ligands at pH 5.0 and pH 7.4.

### Fluorescence ligand binding assays

Fluorescence-based ligand binding assays were performed based on the method described by Sun X. et al. (2016). According to previous studies about the rice-specific volatiles (Fujii et al., 2010; He et al., 2011; Yang et al., 2014; Zhang et al., 2014), 25 potential ligands were selected for the fluorescence binding assays (Table 1). All the ligands used in this study were purchased from Sigma-Aldrich (St. Louis, MO, USA) and stored according to manufacturer instructions. The ligand binding affinity for various ligands was determined by using the 1-NPN (N-phenyl-1-naphthylamine) as a fluorescent probe. RF-5301PC fluorimeter (Shimadzu, Kyoto, Japan) was used for fluorescence binding assay at 25°C with 10 nm slit width and 1 cm light path quartz cuvette for emission and excitation. The 1-NPN/NlugCSP8 mixture was excited using an excitation wavelength of 337 nm, and the fluorescence intensity was recorded between 350 and 600 nm following an established protocol (Ban et al., 2002). The 1-NPN and all the potential ligands were prepared in spectrophotometric-grade methanol. The binding constant for 1-NPN was measured by adding aliquots of 1 mM 1-NPN into a 2 μM solution of protein in 30 mM Tris-HCL at room temperature. To measure the binding affinity of various potential ligands, the 2 μM solution of protein was titrated with 1 mM 1-NPN with the final concentration of each ligand between 0 and 20 μM. For each test, fluorescence measurement was conducted after the reaction was incubated for 2 min at room temperature (Liu et al., 2015). Three independent measurements were used to obtain the binding data. 1-NPN/NlugCSP8 dissociation constants (Kd) were calculated from Scatchard plots of the binding data using the Prism 5 software (GraphPad, La Jolla, CA, USA). The curves were linearized using the Scatchard plot program (Campanacci et al., 2001). The dissociation constants of the competitors were determined by using the corresponding IC_50_-values according to the equation Ki = [IC_50_]/ (1+[1-NPN]/K_1−NPN_), where IC_50_ represents the concentration of ligand which decreases the fluorescence intensity of [1-NPN], [1-NPN] is the free concentration of 1-NPN and K_1−NPN_ is the dissociation constant of the NlugCSP8/1-NPN complex (Ban et al., 2003; Tian and Zhang, 2016). For the reader's convenience, data were recalculated as 1/ki × 1,000, for which a larger value designates a stronger ligand binding affinity.

**Table 1:** Binding affinities of different ligands (long chain and without long chain) to NlugCSP8 evaluated via competitive ligand binding assays by using the fluorescent probe, 1-NPN.

**Ligands**	**CAS No#**	**Purity (%)**	**pH 7.4**		**pH 5.0**	
			**IC_50_ (μM)**	**Ki (μM)**	**IC_50_ (μM)**	**Ki (μM)**
**LIGANDS WITH LONG CHAIN**						
3-Pentanol	584-02-1	98	23.47	19.55	31.43	27.40
Cis-3-hexen-1-ol	928-96-1	97	27.46	22.88	37.96	33.09
Trans-2-hexenal	6728-26-3	97	27.98	23.31	11.57	9.66
Trans-2-hexen-1-ol	928-95-0	98	25.86	21.54	18.19	15.86
Nonadecane	629-92-5	99	17.20	14.33	28.28	24.65
Eicosane	112-95-8	100	14.86	12.37	14.00	12.20
Hexadecane	544-76-3	98	19.05	15.87	27.33	23.83
2-Tridecanone	593-08-8	98	15.02	12.51	11.96	10.43
Hexanal	66-25-1	98	17.28	14.40	10.96	9.56
1-Octen-3-ol	3391-86-4	98	23.26	19.37	22.06	19.23
Dodecyl aldehyde	112-54-9	99	14.64	12.20	32.59	28.42
Nerolidol	7212-44-4	98	12.01	10.01	9.61	8.38
2-Heptanol	543-49-7	99	16.53	13.77	11.20	9.77
Linalool	78-70-6	97	23.43	19.52	36.90	32.17
Farnesene	502-61-4	98	53.81	44.82	198.71	173.26
**LIGANDS WITHOUT LONG CHAIN**						
(–)-Limoonene	5989-54-8	97	24.41	20.35	20.41	17.80
(–)-Terpinen-4-ol	20126-76-5	98	19.01	15.84	11.43	9.97
4-Isopropyl to luene	99-87-6	98	20.28	16.89	22.68	19.77
α-Terpinene	99-86-5	96	16.58	13.81	16.36	14.27
Terpinolene	586-62-9	96	25.59	21.31	31.20	27.20
(+)-3-Carene	13466-78-9	90	32.38	26.98	39.86	34.75
Methyl benzoate	93-58-3	100	23.57	19.63	17.77	15.50
R-(+)-limonene	5989-27-5	98	15.87	13.22	14.40	12.56
α-Terpineol	10482-56-1	97	24.13	20.10	15.49	13.50
Cyclohexanol	108-93-0	99	51.23	42.68	95.49	83.26

### Double-stranded RNA synthesis

The full coding sequence of NlugCSP8 and green fluorescent protein (GFP) were cloned into pMD-18T vector and used as templates for the target sequences amplification. The target sequences of NlugCSP8 and GFP were amplified by RT-PCR using specific gene primers conjugated with 19 bases of the T7 RNA polymerase promoter (Table S1). dsRNA was synthesized from PCR products as templates by using the T7 Ribomax Express RNAi System Kit (Promega, Madison, WI, USA). After synthesis, the dsRNA was precipitated by adding isopropanol and resuspended in nuclease-free water. The purified dsRNAs were quantified spectrophotometrically at 260/280 nm and integrity was examined by agarose gel electrophoresis.

### dsRNA injection and analysis of gene silencing

Microinjector (World Precision Instruments Inc., Sarasota, FL, USA) fitted with a glass capillary needle was used for dsRNA injection assays. BPH was anesthetized using the CO_2_ for 30 s and placed on agarose plate. Prior to injection with dsRNAs, BPH was placed in the groove using a pointed brush. Each individual was nanoinjected with 30 nL of 5 ng/nL dsRNAs into the conjunctive between prothorax and mesothorax under a microscope. For dsCSP8 and dsGFP, 100–150 3rd instar nymphs were injected in every replication and three biological replicates were used. Injected nymphs were placed on fresh rice seedlings to recover, and reared at 26 ± 2°C, humidity 75 ± 5% and 8/16 h dark/light for 1–7 days. The mortality was recorded every day following injection. Six synchronous nymphs were selected randomly at 1st, 2nd, 3rd, 5th, and 7th days after injection for subsequent RNA extraction. The relative mRNA expression levels were determined in the injected group, while others were normalized to one in the non-injection group. All the data were expressed as the mean ± SE of three separate measurements.

### Olfactory behavioral assays

BPH behavior responses to different ligands were tested in an H-tube olfactometer similar to which previously used by Yi et al. (2018) in our laboratory. The H-tube olfactometer mainly consists of two glass tubes (arms) with gauze at its top end. These two glass tubes were connected by another tube (5 cm in diameter, 20 cm long with a hole of 1 cm in the middle for releasing BPH). Twenty macropterous (10 from each sex) BPH adults were introduced into the H-tube and the number of BPH was counted at 30 min after their introduction. Liquid paraffin was used as in control arm. Rubber septa were absorbed in the liquid paraffin and solutions of the odor molecules to be tested (liquid paraffin+ different concentration of tested volatile) and placed at room temperature. After 24 h, one rubber septa from each control and tested volatile was put in each glass arm. After one replication, rubber septa were changed and three treatments (1, 10, and 100 μl/mL) of tested volatiles against macropterous adults were tested in eight replications. After four replications, the H-tube olfactometer was washed with 75% alcohol and the liquid paraffin rubber septa were placed in another arm to complete the other four replications. The impact of NlugCSP8-dsRNA on the preference of *N. lugens* was also tested by H-tube olfactometer assays. Corresponding control experiments without dsRNA injection were performed to investigate whether the preference of *N. lugens* was affected by volatile concentration change. Three treatments of BPH (NlugCSP8-dsRNA injected, GFP-dsRNA injected, and without injection) were tested in four replications. As in case of dsRNA injected insects, the concentration of volatiles that have highly significant attractive results on non-injected insects used for dsCSP8 and dsGFP injected insects. In order to evaluate the best RNAi effect, mRNA levels of NlugCSP8-dsRNA injected insects were determined and compared with the GFP-dsRNA-injected and non-injected insects, prior to H-tube olfactometer bioassay. Based on the findings of previous step, we re-inject the BPH and the individuals with the best post-injection RNAi effect after 7-days, were used in the H-tube olfactometer bioassay. Bioassays were performed under controlled conditions at 26 ± 2°C and 75 ± 5% relative humidity.

### Molecular modeling and ligand docking

Delta-BLAST was performed (NCBI: http://blast.ncbi.nlm.nih.gov/Blast.cgi) with the NlugCSP8 sequence, against the protein data bank (PDB: http://www.rcsb.org) by using the SWISS-MODEL server (SWISS-MODEL: http://swissmodel.expasy.org/). After BLAST resulted sequences having identities > 40% were selected for subsequent analysis and Clustal W (http://embnet.vital-it.ch/software/ClustalW.html) was used for multiple sequence alignment. The top hit protein sequence was selected on the basis of sequence homology, query coverage, phylogeny and the number of Cys (cysteine) residues, and the template of CSPsg4 from *Schistocerca gregaria* (PDB ID: 2GVS_A) was further used to build a 3D model of NlugCSP8 (Tomaselli et al., 2006). Regarding molecular docking studies, a number of docking programs are available; here we used Docking protocol implemented in MOE (MOE, version 2012.10) designed by Chemical Computing Group (Vilar et al., 2008), in order to predict the binding sites of NlugCSP8. The ligands [Nerolidol, Hexanal, Trans-2-hexenal, 2-Heptanol, and (−)-terpinen-4-ol] were chosen to dock into the binding pocket of the 3D structure of NlugCSP8 because these ligands exhibited strong binding affinities with NlugCSP8 in experimental analysis. The default parameters have been used to calculate the interaction of ligand molecules and score against respective ligands (Rescoring 1: London dG, Refinement: Forcefield, Rescoring 2: GBVI/WSA dG, Placement: Triangle Matcher). The most suitable docked ligand-protein structure was designated on the basis of RMSD (Root Mean Square Deviation) values and minimum S-score. The S-score is the value calculated by built-in scoring functions of MOE on the basis of ligand-binding affinity with receptor protein after docking. While, RMSD value is generally used to compare the docked conformation with the reference conformation or with other docked conformation (Wadood et al., 2014; Qamar et al., 2016).

### Statistical analysis

The SPSS (Statistical package for the social sciences) computer software version 22.0 was used for data analysis (SPSS Inc., Chicago, IL). All qRT-PCR data were statistically analyzed using ANOVA (one-way analysis of variance) followed by Tukey's Honestly Significant Difference (HSD) test. *P* < 0.05 was considered statistically significant. The chi-squared test was used to determine significant differences in the number of insects choosing a particular odor.

## Results

### Characterization and homology analysis of the NlugCSP8

**Figure 1:**
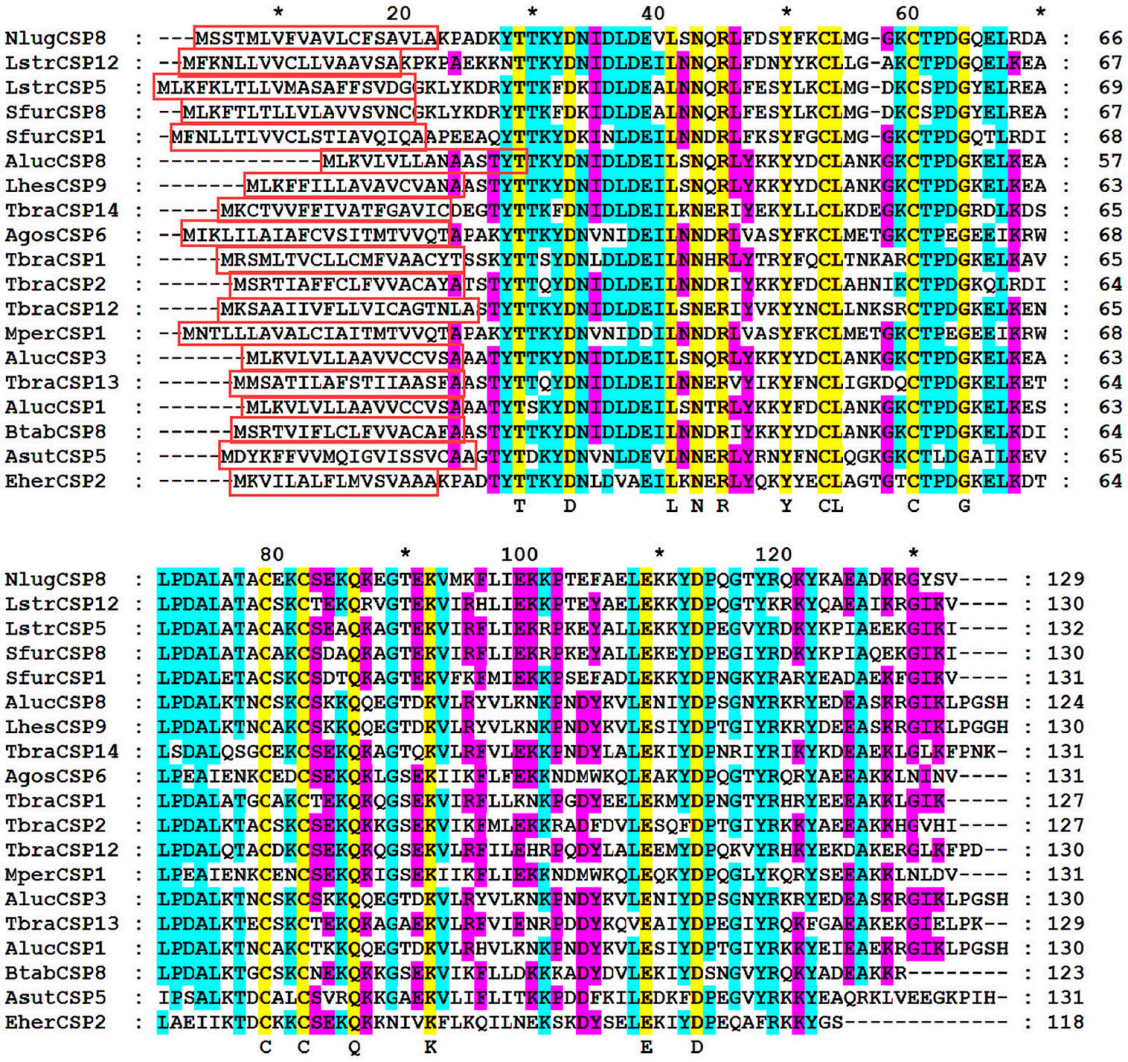
Alignment of NlugCSP8 to orthologous of hemipteran insect species. Predicted signal peptides are boxes. Conserved cysteines and other conserved residues were highlighted by yellow color. The residues are marked with Arabic numbers and ^*^ representing the set of 10 amino acids. The other insect species are: *Apolygus lucorum* (Aluc), *Lygus hesperus* (Lhes), *Triatoma brasiliensis* (Tbra), *Bemisia tabaci* (Btab), *Euschistus heros* (Eher), *Adelphocoris suturalis* (Asut), *Myzus persicae* (Mper), *Aphis gossypii* (Agos), *Laodelphax striatella* (Lstr), and *Sogatella furcifera* (Sfur). GenBank accession number for all CSPs genes are: *AlucCSP1*, KC136232.1; *AlucCSP3*, JN573219.1; *AlucCSP8*, KC136239.1; *LhesCSP9*, KU194356.1; *TbraCSP1*, LT555316.1; *TbraCSP2*, LT555317.1; *TbraCSP12*, LT555327.1; *TbraCSP13*, LT555328.1; *TbraCSP14*, LT555329.1; *BtabCSP8*, KY305451.1; *EherCSP2*, HQ677768.1; *AsutCSP5*, KT347586.1; *AgosCSP6*, KC161568.1; *MperCSP1*, FJ387490.1; *NlugCSP8*, ACJ64054.1; *LstrCSP5*, KC516758.1; *LstrCSP12*, KC516765.1; *SfurCSP1*, KC516736.1; *SfurCSP8*, KC516743.1.

The full-length cDNA encoding NlugCSP8 was cloned and verified by sequencing. It showed 100% amino acid identity with the previously deposited sequence of NlugCSP8 (GenBank accession number: ACJ64054.1) (Xu et al., 2009). NlugCSP8 sequence analysis revealed a full-length Open Reading Frame (ORF) of 390 nucleotides encoding 129 amino acids residues, with an isoelectric point of 6.34 and a molecular weight of 14.6 kDa. At their N-terminus, NlugCSP8 contain signal peptide of 19 residues suggesting the solubility of NlugCSP8 (Figure S1). The sequence alignment of NlugCSP8 and the corresponding CSPs obtained from other hemipteran species was performed (Figure 1). The alignment analysis showed that four conserved cysteines obviously presented in all CSPs. The NlugCSP8 shares the highest identity (50–71%) with other hemipteran CSPs. The highest scoring identities, based on the morphological characters of their phylogenetic interactions, were 71% with *Laodelphax striatella* (LstrCSP12) and 62% with *Sogatella furcifera* (SfurCSP1). The phylogenetic relationship showed that the NlugCSP8 had closer ancestor from the same order of insects. We searched NlugCSP8 for homologs in other insect species using tblastn with an *e*-value cut off 10e-30. The search result revealed that NlugCSP8 possessed sequence homologous to 144 insect CSPs (Figure S2). Among them, there are 52 Hemipteran, 9 Dipteran, 25 Lepidopteran, 55 Coleopteran, 2 Hymenopteran, and 1 Neuropteran CSPs.

### Expression patterns of NlugCSP8

**Figure 2:**
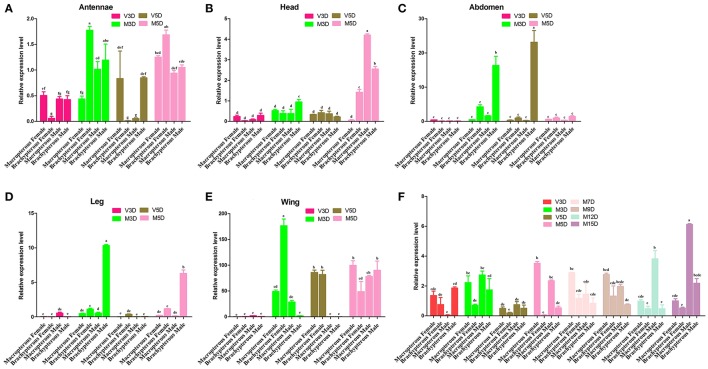
The expression profiles of NlugCSP8. Relative mRNA expression level analyses by qRT-PCR in different tissues of unmated and mated males and females from different developmental stages. The X-axis shows the macropterous female/male and brachypterous female/male and the Y-axis was the relative expression quantity. Total RNA was extracted from **(A)** Antennae; **(B)** Head (without olfactory appendages); **(C)** Abdomen; **(D)** Leg; **(E)** Wing; **(F)** different stages of development. (V) and (M) stand for the virgin and mated insects. The mRNA expression level was normalized relative to the β-actin transcript levels. The different letter on the top of each bar means significant differences (*P* < 0.05). The data indicated the mean values ± SE of three biological replicates.

The qRT-PCR dataset (Figure 2) was based on different tissue samples (At, antennae; H, head; Ab, abdomen; L, leg; W, wing) from unmated and mated BPH at 3 and 5 days old, as well as the whole-body of mated (3, 5, 7, 9, 12, 15 days old) and unmated (3 and 5 day old) insects. The resulted dataset was employed to characterize the pattern of developmental expression of the NlugCSP8 gene in different developmental stages. Transcript levels were also tested in the two different wing forms of males and females. The qRT-PCR results showed that NlugCSP8 was highly expressed in mated brachypterous female antennae with low expression level in unmated brachypterous female antennae (Figure 2A). Significant differences of expression levels were also observed in the head between male and female at 5-day-old mated adults (Figure 2B). Pursuing this further, NlugCSP8 expression level was also higher in the unmated 5-day-old brachypterous male abdomen when compared to the mated 5-day-old brachypterous male abdomen (Figure 2C). On the other hand, NlugCSP8 expression in mated 3 and 5-days-old brachypterous male leg was significantly higher than unmated 3 and 5-days-old brachypterous male leg (Figure 2D). The qRT-PCR results displayed that the levels of NlugCSP8 mRNA were correlated with age and mating status and the gene was highly expressed in mated 3-day brachypterous female wing (Figure 2E). No significant differences were observed between macropterous and brachypterous BPHs of both sexes, except for unmated 3-day-old and mated 5, 9, and 15 days-old BPH (Figure 2F). However, significant differences between male and female expression levels were observed for NlugCSP8 in 15 days-old BPH (for mated adults). For instance, the expression levels of NlugCSP8 in mated 3, 5, and 15 days-old macropterous BPHs were higher than those in brachypterous adults at the same stage (Figure 2F). Closer inspection of the 3-day unmated insects showed that the expression level of NlugCSP8 was significantly higher in brachypterous male than macropterous male. Interestingly, the relative expression in macropterous females was significantly affected by mating status. The expression levels of NlugCSP8 in mated macropterous females were significantly (*P* < 0.05) higher than in unmated macropterous females at 5-day-old BPH (Figure 2F). However, significant differences were also observed between mated and unmated 5-day-old macropterous males (*P* < 0.05). Overall, NlugCSP8 was more highly expressed in mated males and females than in unmated individuals.

### Fluorescence binding assay

**Figure 3:**
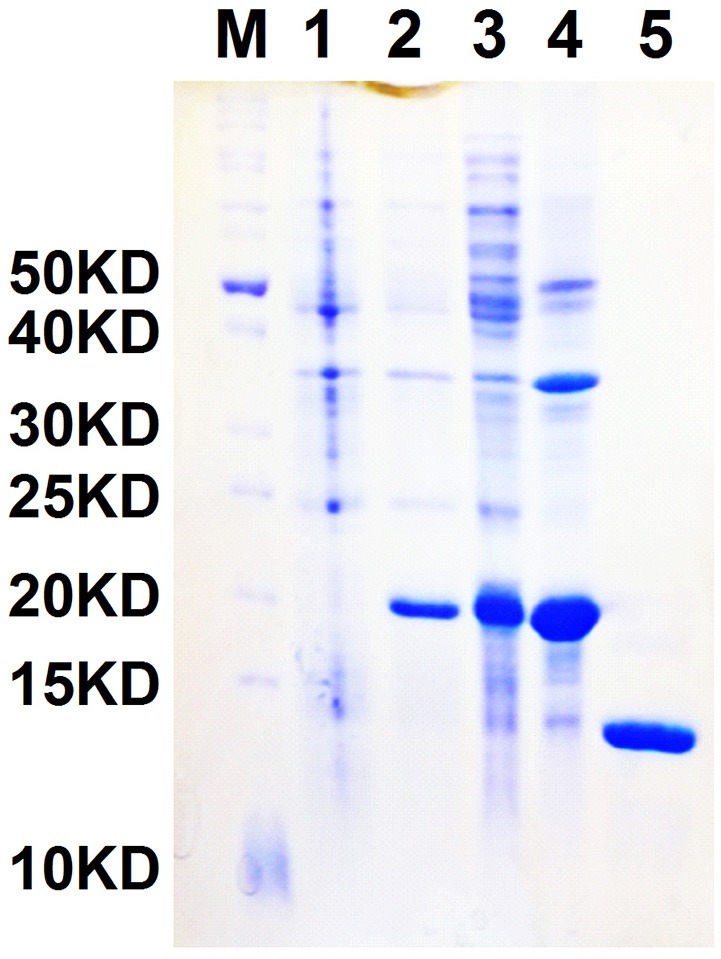
SDS-PAGE analyses showing the expression and purification of recombinant NlugCSP8. Lane M: Molecular marker, Lane 1 and 2: bacterial cells before and after induction by IPTG, respectively, Lane 3: Inclusion body of induced BL21 (DE3) bacteria with pET-30a/NlugCSP8, Lane 4: supernatant of induced BL21 (DE3) bacteria with pET-30a/NlugCSP8, Lane 5: purified protein without His-tag.

**Figure 4:**
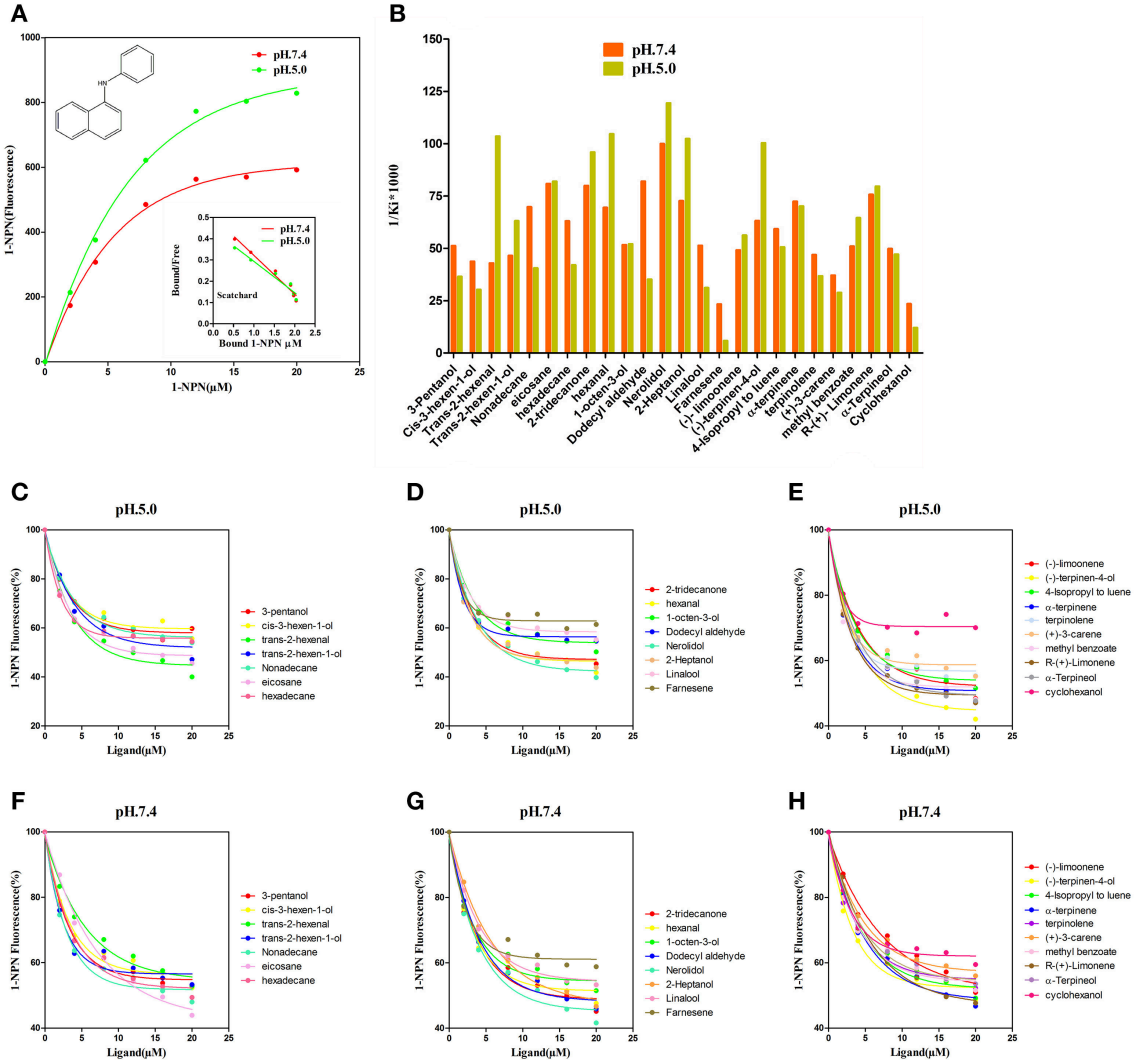
Fluorescence competitive ligand-binding assays of NlugCSP8. **(A)** The binding curves for 1-NPN to NlugCSP8 at pH 5.0 and pH 7.4. A 2 μM solution of NlugCSP8 in 30 mM Tris-HCL buffer (pH 5.0 and pH 7.4) was titrated with a 1 mM 1-NPN solution in spectrophotometric grade methanol to a final concentration of 0–20 μM, and the emission spectrum was recorded between 350 and 600 nm. **(B)** Ligand binding affinity (indicated by 1/Ki^*^1,000) of NlugCSP8 with 25 compounds at pH 5.0 and pH 7.4. **(C,D,F,G)** competitive binding curves of long chain ligands to NlugCSP8 at pH 5.0 and pH 7.4. **(E,H)** competitive binding curves of without long chain ligands to NlugCSP8 at pH 5.0 and pH 7.4. A mixture of the recombinant NlugCSP8 and 1-NPN in 30 mM Tris-HCL (pH 5.0 and pH 7.4) was titrated with 1 mM solution of each competing ligand to the final concentration of 0–20 μM.

NlugCSP8 was successfully expressed using a bacterial system with high recombinant protein yield (about 20 mg/L) as a soluble protein. The recombinant protein was then purified by passing it through a Ni-ion affinity chromatography column. The His-tag was cleaved off with recombinant bovine enterokinase (Figure S3). The expression and purification of the recombinant protein were assessed by 15% SDS-PAGE (Figure 3). The fluorescence binding assays were performed using the fluorescent probe N-phenyl-1-naphthylamine (1-NPN) as a reporter. First, NlugCSP8 titration with increasing concentration of 1-NPN, saturated and linear Scatchard plots were observed at pH 7.4 and pH 5.0, with a dissociation constant of 4.99 and 6.80 μM, respectively (Figure 4A). A fluorescence competitive binding assay of NlugCSP8 with long chain and without long chain compounds using 1-NPN as a fluorescent probe was performed (Table 1). Considering the different mechanisms of ligand-binding and release in CSPs/OBPs, we used pH 7.4 and pH 5.0 in order to simulate the pH environment and dynamic changes in the body *in vitro*. Figure 4B compares the binding values of ligands at both pH-values. The comparison indicated that the ligands displayed higher binding affinities at pH 5.0 (Figure 4B). The most striking results to emerge from data is the broad binding properties of NlugCSP8 toward most of host plant-derived volatiles emitted from rice. These results demonstrated that NlugCSP8 achieved the highest binding affinities with nerolidol, hexanal, trans-2-hexenal, and 2-heptanol (Ki < 10) at pH 5.0 (Figures 4C,D) and pH 7.4 (Figures 4F,G). In the same vein, the NlugCSP8 displayed high binding affinities with (−)-terpinen-4-ol (Ki < 10) at pH 5.0 (Figure 4E), and with R-(+)-Limonene at pH 7.4 (Figure 4H). However, NlugCSP8 exhibited weak binding affinity to cyclohexanol and farnesene (Ki > 40 μM) at pH 7.4 and pH 5.0. Taken together, these results also suggest that there is a relationship between the binding affinity of NlugCSP8 and carbon chain length of ligands. In particular, long chain ligands exhibited a higher binding affinity as compared with shorter chain ligands. For example, nerolidol with a backbone of 12 carbon atoms exhibited the strongest binding affinity to NlugCSP8 at pH 5.0, followed by hexanal, trans-2-hexenal, and 2-heptanol with backbones of 6, 7, and 7 carbon atoms, respectively.

### Behavioral trials

**Figure 5:**
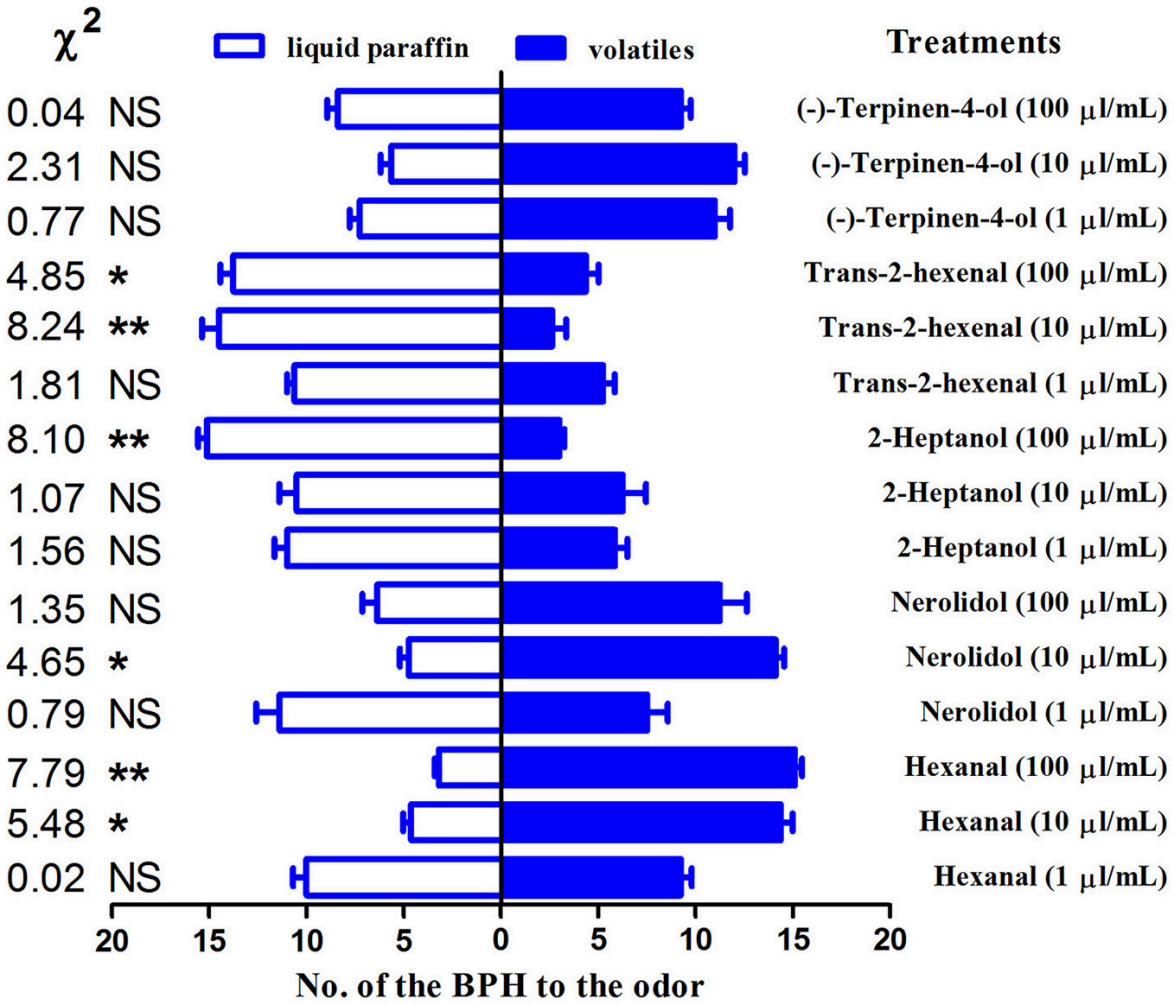
H-tube olfactometer test of the response of *Nilaparvata lugens* to compounds. The number (mean ± SE, *n* = 8) of macropterous BPH male and female adults in H-tube tests between liquid paraffin (control) and different odorant chemicals. Chi-squared test was used to evaluate the significant differences in the number of insects choosing a particular odor. ^*^indicates a significant difference (*P* < 0.05), and ^**^indicates a highly significant difference (*P* < 0.01). ^*NS*^indicates no significant difference.

The behavioral responses to the 5 compounds that exhibited high binding affinities (Ki < 10 μM) for the NlugCSP8 were tested in an H-tube olfactometer. Four compounds out of five were able to elicit behavioral responses in *N. lugens* (Figure 5). Contrasting responses were also observed in chemical compounds that modulate behavior due to concentration-dependent effect. BPHs displayed repellency when the concentration of hexanal was 1 μl/mL, while it strongly behaved as attractant at 100 μl/mL. Such attraction became weakened at 10 μl/mL. Nerolidol showed a significant attraction to BPHs at a concentration of 10 μl/mL. However, the BPHs showed significant aversion to 2-heptanol and trans-2-hexenal, while (−)-terpinen-4-ol was attractive at concentrations of 1 μl/mL and 10 μl/mL with no significant effect on insect's behavior.

### Behavioral analysis after NlugCSP8 mRNA expression profile silencing by dsRNA

**Figure 6:**
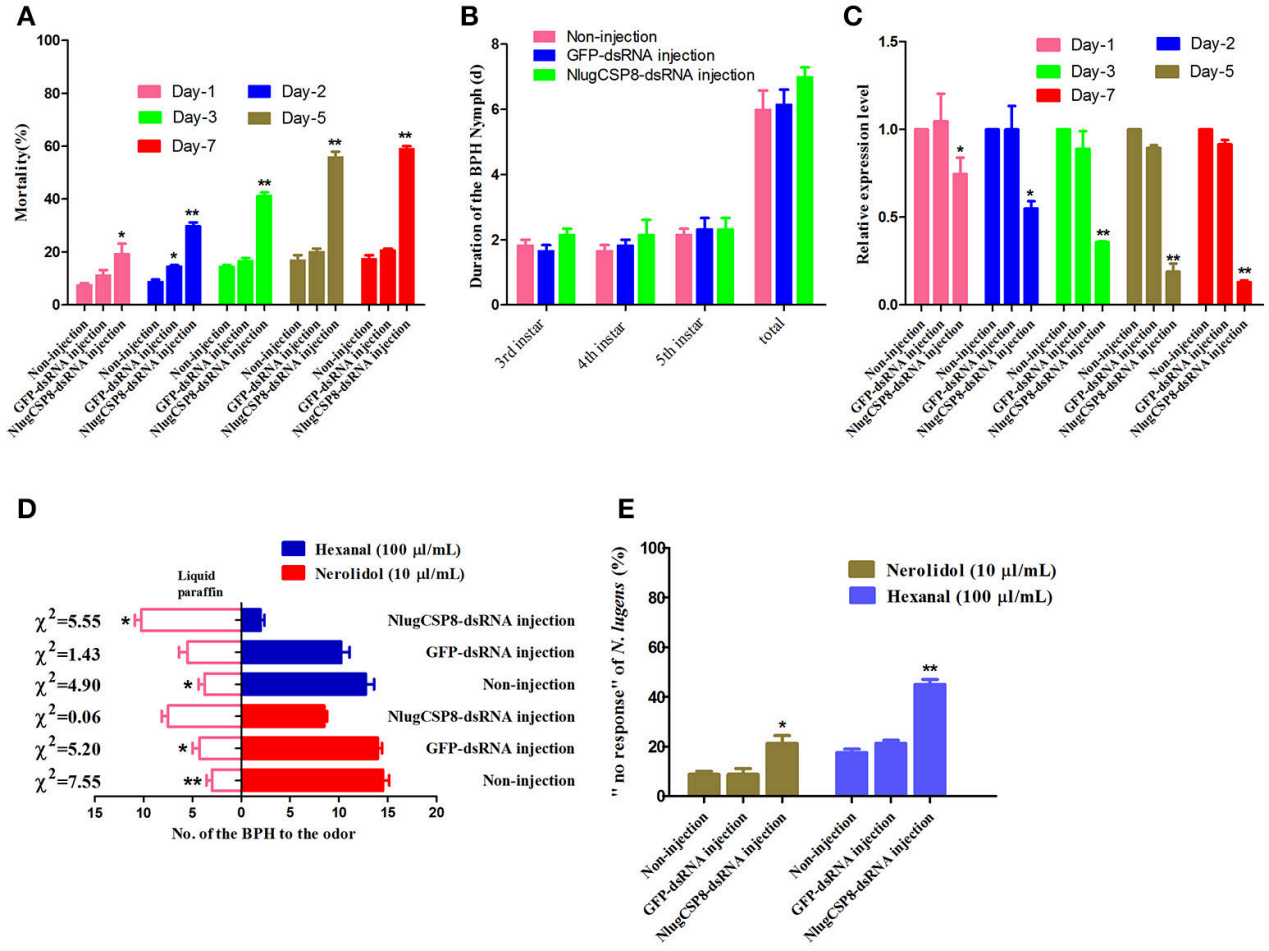
RNA interference injection with dsRNA of NlugCSP8 and phenotype changes after gene silencing. **(A)** *N. lugens* mortality when injected on dsRNA at different kinetic points. Mortality was recorded on daily basis. **(B)** The duration of BPH nymph (mean ± SE, *n* = 5) of 3rd instar, 4th instar, 5th instar, and the total duration from 3rd to newly emerged adult on without-injection, dsRNA-GFP and dsRNA-CSP8 injected insects. **(C)** Analyses of mRNA transcript levels of NlugCSP8 after dsRNA injection. β-actin was used as an internal reference gene. The results were evaluated using a 2^−ΔΔCT^ method, and the 2^−ΔΔCT^ value of calibrant equals to 1.0. Asterisks on the top of the bars specify that the values were significantly different (^*^P < 0.05; ^**^P < 0.01, Tukey's *t*-test, *n* = 3). **(D,E)** Behavioral responses and non-responding *N. lugens* recording from post-RNAi injection. Concentration of selected ligands: 10 μl/mL (nerolidol), 100 μl/mL (hexanal) and *n* = 4; mean ± SE. Liquid paraffin was used as a control in this study.

To determine the function of NlugCSP8 *in vivo*, dsRNA against *N. lugens* (NlugCSP8) were injected into 1-day-old third-instar nymphs. At the seventh day, the average mortality of the nymphs injected with the dsCSP8 and dsGFP increased to 55.85 and 20%, respectively (Figure 6A). The durations of three nymphal instars (N3-N5) were not affected by dsRNA-NlugCSP8 injection (Figure 6B). In addition, no significant differences were observed in the mRNA levels of the target gene between non-injected and dsGFP injected groups. NlugCSP8 expression was significantly reduced by 25.5% in 1 day after injection with 150 ng dsCSP8 (Figure 6C). Compared with the control group that received dsCSP8 against green fluorescent protein (dsGFP), the maximum reduction of 86±1.01% occurred at the 7th day (Figure 6C).

To explore the possible impact of NlugCSP8 knockdown, we conducted initial behavior screening to identify chemical compounds that may elicit behavior response in BPH. We identified two selected compounds that elicited the strongest attractive responses from BPH (Figure 5). For hexanal, behavior response was reduced significantly in RNAi treated insects, as compared with controls (Figure 6D). On the other hand, the behavioral activity of nerolidol was sharply reduced in the knock-down BPHs and the attraction activity was completely lost in the insects injected with dsCSP8. However, the ratio of “no response” BPHs in dsCSP8 group was also significantly increased compared to dsGFP and non-injection control group (Figure 6E).

### Three-dimensional structure modeling and molecular docking

**Figure 7:**
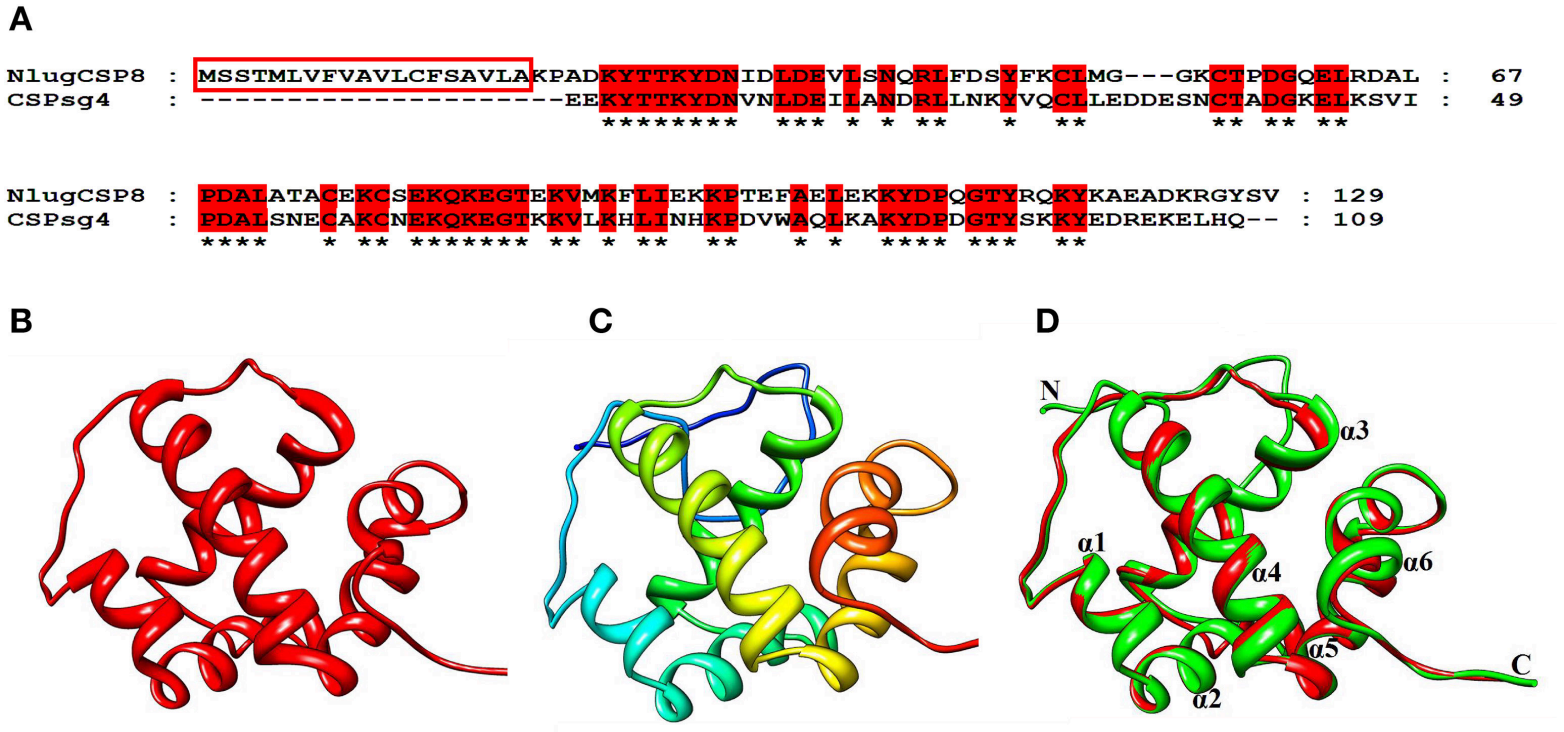
Three-dimensional structural modeling of the NlugCSP8. **(A)** Sequence alignment of NlugCSP8 and CSPsg4. In the alignment of the two proteins, NlugCSP8 signal peptides are boxed and conserved residues are highlighted in red. **(B)** 3D structure of CSPsg4 selected as a template (PDB ID: 2GVS_A). **(C)** Predicted 3D structure of *N. lugens* encoded chemosensory protein 8 (ACJ64054.1). **(D)** Superimposed structure of NlugCSP8 and the template CSPsg4. The predicted models of NlugCSP8 and template structure of CSPsg4 are shown in green and red, respectively. Six α-helixes, N-terminal (N), and C-terminal (C) are marked.

The NlugCSP8 sequence was compared to all known proteins in the Protein Data Bank (PDB) and the results revealed that chemosensory protein sg4 from *S. gregaria* (CSPsg4) (PDB ID: 2GVS_A) achieved the highest sequence similarity (54%) with NlugCSP8 and it was selected as a template to model the 3D structure of the NlugCSP8 (Figures 7A,B). From the results of homology modeling, the best model (Figure 7C) was selected on the basis of RMSD-value (0.34Å) and its quality was further checked by Ramachandran Plot on the basis of φ and ψ-values constrained in specific areas (Figure S4). Ninety-one of residues were found in the favored region which highlights the quality of a predicted model and plot also showed a larger number of residues found in α-helices region (Figure S4). The results of the predicted 3D structure showed that NlugCSP8 is an α-helix-rich globular protein that consists of six α-helices: α1 (residues Leu34–Ser39), α2 (residues Gln41–Met52), α3 (residues Pro58–Ala72), α4 (residues Glu80–Lys96), α5 (residues Pro98–Tyr108), and α6 (residues Arg115–Ala122) and contains multiple hydrophobic cavities, which could be involved in ligand binding. Evaluation of structure and superimposition of selected model with the template also exhibited that it consists of six α-helices with a very low RMSD-value of 0.34Å. The RMSD-value 0.34Å indicates that both template and NlugCSP8 protein have similar folds. It also further supports the idea that the complete confirmation of the modeling target was very similar to that of the template (Figure 7D).

**Figure 8:**
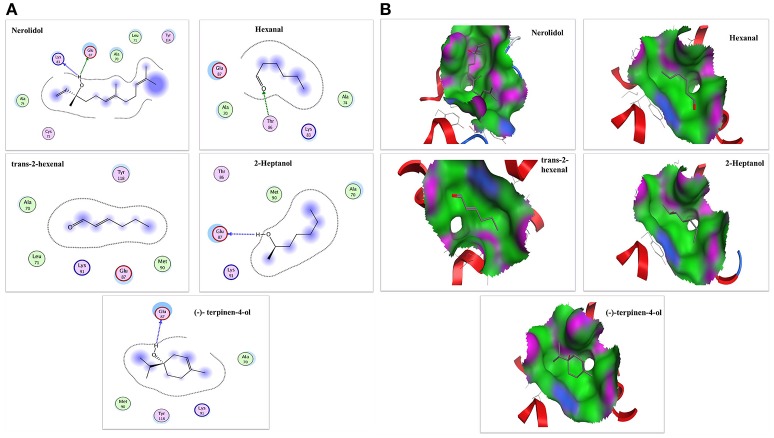
The interaction diagram and binding models of NlugCSP8. **(A)** 2-Dimensional predicted hydrogen-bond interaction view with NlugCSP8 residues by molecular docking. The red and green amino acids represent polar and non-polar, respectively. The dashed lines with arrows express the expected hydrogen-bond interaction. **(B)** Binding pocket mode of ligands inside active site of NlugCSP8. The red area represents hydrophilia and green area represents hydrophobicity. The blue atom expresses nitrogen atom. The red atom expresses oxygen atom.

To confirm the results of our ligand binding assay and provide insight into the mechanism of NlugCSP8 interaction with host compounds, molecular docking of five selected compounds [Nerolidol, Hexanal, Trans-2-hexenal, 2-Heptanol, and (−)-terpinen-4-ol] was performed. The protein binding sites and functional residues interacting between the NlugCSP8 and ligands are presented in Table 2. The residues identified by current docking simulations, including Lys83, Thr86, and Glu87 were the main participants for NlugCSP8, whereas residues including Ala70, Leu71, Aal74, Cys75, Met90, Lys91, Tyr114, and Tyr118 had a close relationship with NlugCSP8. Figure 8A shows the interaction model of the NlugCSP8 and different compounds with some potential residues. As far as NlugCSP8 is concerned, there are 5 amino acid residues (Ala70, Leu71, Ala74, Cys75, and Tyr114) that may interact with nerolidol. Glu87 and Lys83 could form a hydrogen bond (H-bond) with the nerolidol. Similarly, hexanal, 2-Heptanol, and (−)-terpinen-4-ol, which also showed strong binding to NlugCSP8, formed H-bond with NlugCSP8. The docking results displayed that the selected compounds could tightly bind toward the center of the NlugCSP8 pocket and influence its activity. In the same vein, the docking result of selected ligands presented a tunnel formed in the NlugCSP8 core and all five ligands docked at the same binding site, where all interactions between the ligands and protein involved residues from helices α3, α4, α5, and α6 (Figure 8B).

**Table 2:** Docking score and molecular docking results of selected ligands.

**PubChem IDs**	**Ligands**	**S-score**	**RMSD**	**Residues interacting with H-bonding**	**Closer contact interacting residues**
5284507	Nerolidol	−18.4272	1.9996	Glu87, Lys83	Ala70, Leu71, Ala74, Cys75, Tyr114
6184	Hexanal	−17.3278	1.6293	Thr86	Ala70, Ala74, Lys83, Glu87
10976	2-Heptanol	−16.0020	1.7858	Glu87	Thr86, Ala70, Met90, Lys91
11230	(-)-Terpinen-4-ol	−15.0213	2.3815	Glu87	Ala70, Met90, Lys91, Tyr118
5281168	Trans-2-hexenal	−13.1066	0.4554	–	Ala70, Leu71, Glu87, Met90, Lys91, Tyr118

## Discussion

CSPs are pervasive and play pivotal roles in the survival and reproduction of arthropods (Pelosi et al., 2014). CSPs are responsible for capturing outside odorants and transport them to the olfactory receptors which are crucial for the development of an olfactory system of insects (Leal, 2013; Li et al., 2015). In insects, the number of CSPs genes ranges from 4 in *D. melanogaster* to almost 70 in *L. migratoria* indicating the number of CSPs genes variability in insect species (Zhou et al., 2013). In this study, we cloned a chemosensory protein (NlugCSP8) from the BPH, and NlugCSP8 has four conserved cysteine C1-C4, which is typical of CSPs and is shared by many other species (Cao et al., 2014; Wang et al., 2016; Xue et al., 2016). NlugCSP8 shares highest identity with CSPs from other insects, possesses the CSP common signature of low molecular mass, an isoelectric point between 5 and 6 and four conserved cysteine residues that conform to the CSP common cysteine sequence spacing pattern (Picimbon et al., 2000). We also identified other amino acid residues that are completely conserved between the examined sequences and NlugCSP8 with four conserved cysteines. The alignment of NlugCSP8 with these CSPs may support the hypothesis that CSPs are highly conserved as they share sequence identity even between CSPs from different insect species (Wanner et al., 2004), and infer important functions that might play role in insect physiology (Gu et al., 2012). In accordance with early research that CSPs had closer ancestry from the identical species, showing CSPs diversification within an order may have curtailed from duplications inside that order (Kulmuni and Havukainen, 2013).

The analysis of relative expression level in different tissues showed that NlugCSP8 is expressed in all of the tissues, indicating that NlugCSP8 has a broad tissue expression profile in *N. lugens*. These results also support the hypothesis that CSPs genes are expressed not only in the antennae as the main olfactory organ, but also in various parts of the insect body, such as the legs, head, thorax, proboscis, pheromone gland, and wings (Wanner et al., 2004; Zhang and Lei, 2015). In particular, some CSP-like genes have been reported to be precisely expressed in the antennae (Calvello et al., 2005), while the NlugCSP8 expression is mainly enriched in wing, abdomen and leg tissues. So far, many CSPs were expressed in different parts of insect body, and some were even expressed in non-chemosensory organs (Jacquin-Joly et al., 2001). For instance, BmorCSP10 from *Bombyx mori* are proposed to be involved in contact chemoreception. Expression of BmorCSP10 is more highly in contact organs (antennae, wings, and legs) than in noncontact organs (head, thorax, and abdomen) (Gong et al., 2007). However, the NlugCSP8 expression is also detected in contact organs (antennae, wings, and legs) and availability of olfactory sensilla on these contact organs, it is anticipated that NlugCSP8 may take part in contact chemoreception, recognizing, and transporting semiochemicals. The CSP from *L. migratoria* (LmigCSP-II) was highly expressed in the sensilla chaetica of the wings and assumed to be involved in contact chemoreception (Zhou et al., 2008). In the western flower thrips, *Frankliniella occidentalis*, the *FoccCSP* was mainly expressed in the antennae and leg tissues and reported to be involved in transporting semiochemicals or some hydrophobic molecules from the lymph to chemosensory receptors (Zhang and Lei, 2015). In this study, NlugCSP8 was highly expressed in the male abdomen and very weakly expressed in abdomen of females, which strongly suggests that this CSP is associated with the reproduction events in *N. lugens* males. Similarly, NlugCSP1 expression in non-olfactory male abdomen also suggested that it might be involved in reproduction process of *N. lugens* (Yang et al., 2014). Additionally, BPH CSPs such as NlugCSP11 are highly expressed in wings and abdomen. A possible explanation for this finding might be that these proteins are involved in gustatory functions, BPH metamorphosis and determination of oviposition and feeding sites (Zhou et al., 2014). In insects, adult female normally does not automatically oviposit at spawning sites, but first examines the appearance of spawning sites through the tarsal sensilla (Thompson, 1988). The high expression of NlugCSP8 in the wing, leg, and abdomen infers that it might be involved in the attraction activity of BPHs adult toward the potential host, which allows the insect to determine the feeding or oviposition site based on the evaluation of the leaf surface using their abdomens or legs (Higashiura, 1989). Another relationship was found between the levels of NlugCSP8 mRNA and age or mating status. It is commonly been assumed that the peak mating is on day 3, and peak laying is on day 5 (Thompson, 1988). Based on these findings, the transcripts of NlugCSP8 were tested from peak mating and peak laying stage of adults. The high level of NlugCSP8 expression in antennae and wings of mated brachypterous females on day 3 might reflect the role of NlugCSP8 in mate seeking behavior and it may also have something to do with gustatory functions because insects wings play somewhat gustatory roles (Xu et al., 2009). The NlugCSP8 expression levels in antennae were more closely related to mating status as compared to age. The observed increase of NlugCSP8 expression level from M3D to M5D in the macropterous female antennae and wings provides further support that this gene might be involved in finding oviposition sites, because day 5 belongs to the peak laying in BPH (Thompson, 1988). A positive correlation was also found between mating behavior and CSP expression level in *N. lugens* (Zhou et al., 2014). In the same way, NlugCSP8 was more highly expressed in mated males and females than in unmated individuals. This high expression after mating may provide evidence that NlugCSP8 plays an important role in the chemoreception of *N. lugens*. Therefore, we focused on the binding characteristics of NlugCSP8 and their relationship with volatiles.

In order to study the functions of NlugCSP8, a total of 25 compounds, mainly rice plant volatiles (Lou et al., 2005; Yang et al., 2009; Fujii et al., 2010), were selected for the fluorescence binding assay at pH 5.0 and pH 7.4. There is some evidence to suggest that nerolidol is a well-known component of rice plant volatile (Hernandez et al., 1989; Yan et al., 2010). In our study, nerolidol showed high binding affinity with NlugCSP8 with Ki-values of 10.01 and 8.38 μM at pH 7.4 and pH 5.0, respectively. The high binding affinity between NlugCSP8 and the plant volatile nerolidol supports the hypothesis that NlugCSP8 may play olfactory roles through binding and transporting the plant volatiles. On the other hand, green leaf volatile hexanal was the most abundant volatile of rice and produced high Electroantennogram response in BPH and some other insects from Hemiptera (Hernandez et al., 1989; Youn, 2002). As expected, NlugCSP8 could bind hexanal, although the Ki was 9.56 μM at pH 5.0. Similarly, 2-tridecanone volatile, also isolated from rice plants, was able to attract BPH (Obata et al., 1983). In our experiments, 2-tridecanone also showed relatively high binding affinities to NlugCSP8, which produced Ki-values of 12.51 and 10.43 μM at pH 7.4 and pH 5.0, respectively. This outcome is contrary to that of Yang et al. (2014) who found that 2-tridecanone possessed relatively weak binding affinity with NlugCSP7. However, to date, functional research of CSPs protein levels in Delphacidae is rare, except for the previous report of Yang et al. (2014) on CSP7 in *N. lugens*. In this report, nerolidol and hexanal also exhibited weak affinities to NlugCSP7, while both strongly bound and showed attraction activity for BPH in case of NlugCSP8 in our study. An interesting finding is that the binding activity of NlugCSP8 also depends upon chain length of ligands. Ligands with long chain exhibited a higher binding affinity as compared with the ligands without chain. Most of the volatiles with relative higher binding ability are compounds with 6–12 carbon atoms. Therefore, carbon chain length appears to affect the binding of NlugCSP8 with ligands. These results match those observed on ligand bindings of SinfCSP19 in earlier studies (Zhang et al., 2014). Nerolidol, with 12 carbon atoms, displayed the highest binding affinity which was in agreement with findings of Zheng et al. (2016) on BhorOBPm2. To support the achievement of these results, molecular modeling and ligand docking were performed. The available 3D structure of NlugCSP8 indicated that it displayed conserved structural features, such as the presence of six α-helices and an internal cavity (Lartigue et al., 2002). The constructed 3D structure of NlugCSP8 is very similar to other previously known insect CSP structures. Like the CSPsg4 of the *S. gregaria* and the CSPMbraA6 of the *Mamestra brassicae*, the CSP8 from *N. lugens* also featured a hydrophobic binding pocket, and the ligand binding differences may be due to some specific amino acids located in the hydrophobic region (Tomaselli et al., 2006). For example, in the CSPsg4, the Trp83 and Ile76 are involved in the binding of oleamide (Tomaselli et al., 2006), while in the CSPMbraA6, the Tyr26 plays an important role in 12-bromo-dodecanol binding (BrC12OH) (Campanacci et al., 2003). Hence, the molecular docking analysis in our study identified several residues, including Lys83, Thr86, Glu87, Ala70, Leu71, Aal74, Cys75, Met90, Lys91, Tyr114, and Tyr118 that may be essential in the binding of volatile compounds by NlugCSP8. These amino acid residues, located in the putative binding pocket of NlugCSP8, may be involved in the recognition and binding of hydrophobic ligands. Pursuing this further, modeling suggested that NlugCSP8 interacts with nerolidol, hexanal, 2-heptanol, and (−)-terpinen-4-ol in order to form H-bonds. Based on these results, we propose that some key residues may be crucial in the interaction of NlugCSP8 with these compounds. Despite these promising results, questions remain on site-directed mutagenesis to assess the function of these residues.

To further support the results of the binding assays, the behavior responses were measured. Four out of five compounds tested elicited a significant behavioral response from *N. lugens*. The compound with high binding affinity to NlugCSP8 did not elicit significant behavior response, signifying that high binding ability *in vitro* doesn't mean high behavioral activity *in vivo*. These behavioral outcomes could be contributed in understanding the sensitivity of insects olfaction related to plant volatiles and may provide strategies for the control of insect pest through identification of semiochemicals responsible for repulsion or attraction of specific insect (Das et al., 2013).

As mentioned earlier, NlugCSP8 probably has different functions related to the finding of oviposition sites, locating suitable mates in addition to olfaction. Thus, RNAi injection experiments against NlugCSP8 were conducted. In the previous study, RNAi technology has been effectively used in BPH, through injection (Liu et al., 2010). Hexanal and nerolidol were identified as strong attractants prior to dsRNA treatment. H-tube olfactometer bioassays of dsRNA-treated BPH revealed that two-choice behavior of BPH was significantly inhibited in hexanal and the attraction activity of nerolidol were lost in insects after silencing NlugCSP8 expression. Based on these findings, we concluded that NlugCSP8 is the pivotal recognition protein for hexanal and nerolidol. Latest studies also recognized that the participation of genes in olfactory functions could be eventually addressed by silencing single genes encoding CSPs or OBPs to influence odor preferences and weaken olfactory performance (Pelletier et al., 2010). However, the ratio of no response BPH also increased in NlugCSP8-dsRNA injected insects as compared to the non-injected control group. These facts support the assumption that NlugCSP8 is involved in behavioral responses, which are the main steps of olfactory reception. Further functional and molecular analysis of other CSPs will provide an exciting opportunity to advance our understanding of olfaction against this monophagous insect and contribute to the development of more efficient and eco-friendly BPH control strategies.

In conclusion, we cloned *CSP8* gene from *N. lugens*. The findings from this study make several contributions to the literature. First, the NlugCSP8 might be involved in finding oviposition sites and locating suitable mates. Second, NlugCSP8 may contribute in binding, transporting, and recognizing plant volatiles. Third, hydrophobic interaction and hydrogen bond play significant roles in the ligand-binding specificity of NlugCSP8 and provide a detailed and reliable olfactory map of chemosensory-protein interaction. Fourth, the reduction in NlugCSP8 transcript abundance leads to a decrease in the behavioral responses to representative attractants. Taken together, these consequences suggest that NlugCSP8 is likely to contribute as a mediator for the responses of *N. lugens* adults to plant volatile attractants.

## Author contributions

The experimental plan conceived and designed by MW, LH, and M-QW. The experiments performed by MW. The data processed and analyzed by MW, AY, MuQ, SA, AA, LH, HA, and M-QW. Writing and editing manuscript MW, F-FZ, AA, AY, HA, MuQ, and M-QW.

### Conflict of interest statement

The authors declare that the research was conducted in the absence of any commercial or financial relationships that could be construed as a potential conflict of interest.

## Supplementary material

Table S1 The primers used in the qRT-PCR, dsRNA synthesis, and protein expression.

Figure S1 Nucleotide and deduced amino acid sequence analysis of NlugCSP8. The predicted putative signal peptides are underlined and denoted by red color. The four conserved cysteine residues are showed in red boxes. The stop codon is indicated with an asterisk.

Figure S2 Molecular phylogenetic analysis of amino acid sequences by neighbor-joining (NJ) method. The tree was constructed using the neighbor-joining method with bootstrap support values (%) based on 1,000 replicates. NlugCSP8 are marked with a solid red circle and all other CSPs from Hemipteran are marked with solid Yellow circles. CSPs from Coleoptera, Lepidoptera, Diptera, Hymenoptera, and Neuroptera are marked with Navy, Lime, Aqua, Blue and Silver circles, respectively. All sequences are available from the NCBI database. Species abbreviations are included for taxon identifications. Dpon (*Dendroctonus ponderosae*), Tcas (*Tribolium castaneum*), Bhor (*Batocera horsfieldi*), Cbow (*Colaphellus bowringi*), Tmol (*Tenebrio molitor*), Malt (*Monochamus alternatus*), Paen (*Pyrrhalta aenescens*), Pmac (*Pyrrhalta maculicollis*), Dhel (*Dastarcus helophoroides*), Agos (*Aphis gossypii*), Mper (*Myzus persicae*), Rdom (*Rhyzopertha dominica*), Cqui (*Culex quinquefasciatus*), Csty (*Calliphora stygia*), Dant *(Delia antiqua*), Apis (*Apis mellifera*), Sinf (*Sesamia inferens*), Harm (*Helicoverpa armigera*), Sexi (*Spodoptera exigua*), Bmor (*Bombyx mori*), Cmed (*Cnaphalocrocis medinalis*), Ofur (*Ostrinia furnacalis*), Cfum (*Choristoneura fumiferana*), Nlug (*Nilaparvata lugens*), Psol (*Phenacoccus solenopsis*), Btab (*Bemisia tabaci*), Adis (*Athetis dissimilis*), Slit (*Spodoptera litura*), Cpal (*Chrysopa pallens*), Lory (*Lissorhoptrus oryzophilus*), Bdor (*Bactrocera dorsalis*), Sfur (*Sogatella furcifera*), Lstr (*Laodelphax striatella*), Alin (*Adelphocoris lineolatus*), Asut (*Adelphocoris suturalis*), Aluc (*Apolygus lucorum*), Eher (*Euschistus heros*), Lhes (*Lygus hesperus*), Tbra (*Triatoma brasiliensis*), Acor (*Anomala corpulenta*), Dcor (*Drosicha corpulenta*), and Hpar (*Holotrichia parallela*).

Figure S3 SDS-PAGE analyses showing the expression and cleavage of recombinant NlugCSP8. Lane M: Molecular marker, Lane 1: Non-induced BL21 (DE3) bacteria with pET-30a, Lane 2–4: different IPTG concentrations used to induce recombinant protein (2, 4, 6 mM from lanes 2 to 4), Lane 5: Eluted protein before cleavage, Lane 6: cleaved protein by the recombinant enterokinase.

Figure S4 The Ramachandran map for the model of NlugCSP8.
