# Supplementary material for: Silencing of Chemosensory Protein Gene NlugCSP8 by RNAi Induces Declining Behavioral Responses of Nilaparvata lugens
Source: Front Physiol. 2018 Apr 12;9:379. doi: 10.3389/fphys.2018.00379 (PMC5906745; doi:10.3389/fphys.2018.00379)
Supplement: Table S1 — The primers used in the qRT-PCR, dsRNA synthesis, and protein expression. [file Table1.DOCX]

**Table S1:** The primers used in the qRT-PCR, dsRNA synthesis and protein expression in this study

| **Purpose/Primer names** | **Primer sequences (5´to 3´)** |  |
| --- | --- | --- |
| **For qRT-PCR** |  |  |
| NlugCSP8-F | TTTTGTGGCGGTTTTGTGCT |  |
| NlugCSP8-R | CCACCCATCAGGCACTTGAA |  |
| **For dsRNA synthesis** |  |  |
| NlugCSP8-F | *TAATACGACTCACTATAGG*ATGTCGTCGACTATGTTG |  |
| NlugCSP8-R  GFP-F  GFP-R | *TAATACGACTCACTATAGG*CTAGACGCTGTAGCCTCT  *TAATACGACTCACTATAGG*AAGGGCGAGGAGCTGTTCACCG  *TAATACGACTCACTATAGG*CAGCAGGACCATGTGATCGCGC |  |
| **Actin gene from *Nilaparvata*** |  |  |
| β-Actin-F | ACTCCGGTGATGGTGTCTCT |  |
| β-Actin-R | GTCGGTCAAGTCACGACCA |  |
| **For protein expression** |  |  |
| **CSP8** |  |  |
| NlugCSP8-F | CCGGAATTCATGTCGTCGACTATGTTGGTTT |  |
| NlugCSP8-R  F, Forward R, Reverse,  GFP, Green Fluorescent Protein | CCGCTCGAGCTAGACGCTGTAGCCTCTCTTG  Restriction sites are underlined  T7 Promoter are italic |  |
