# Supplementary figures and images for: Silencing of Chemosensory Protein Gene NlugCSP8 by RNAi Induces Declining Behavioral Responses of Nilaparvata lugens
Source: Front Physiol. 2018 Apr 12;9:379. doi: 10.3389/fphys.2018.00379 (PMC5906745; doi:10.3389/fphys.2018.00379)

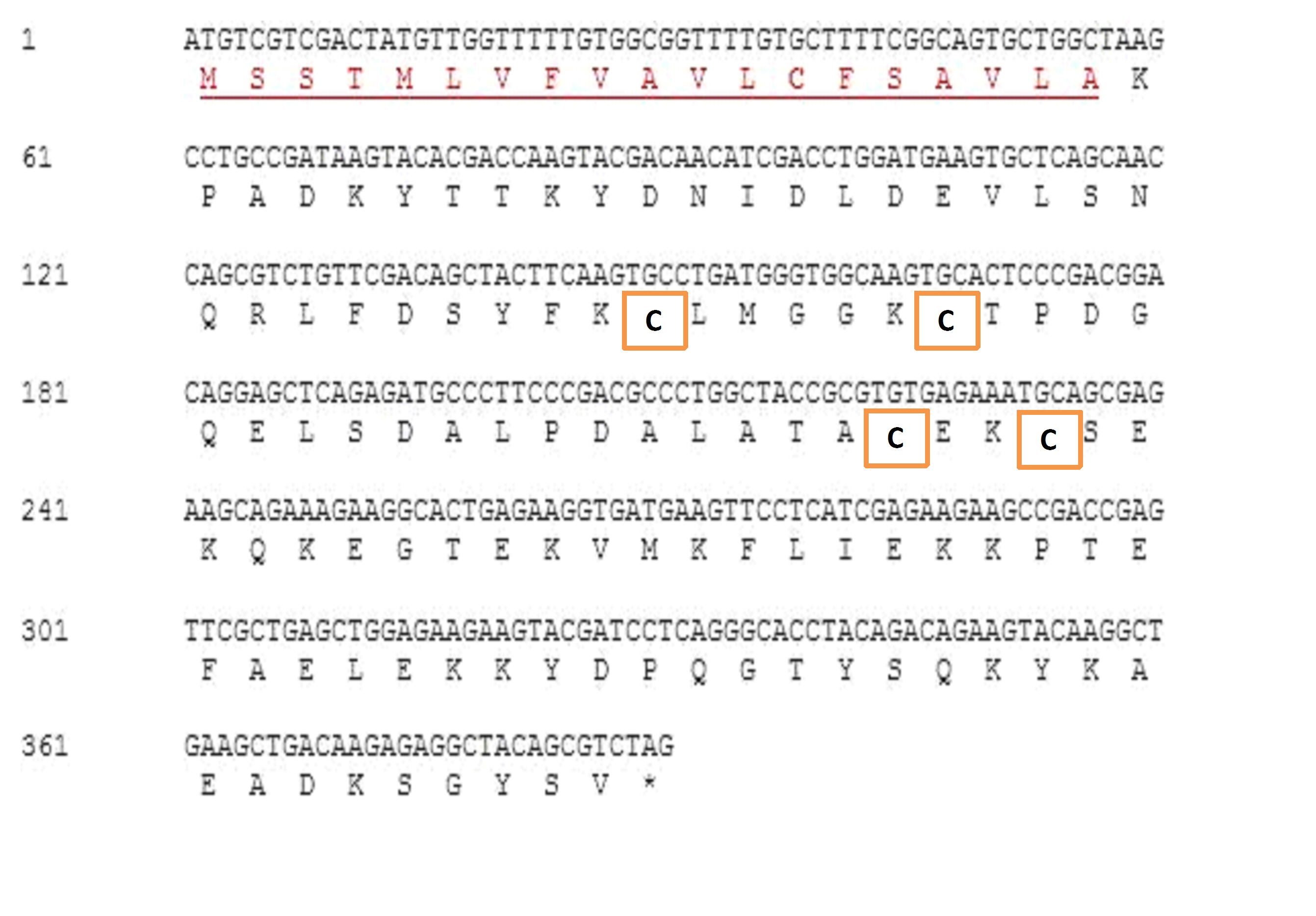

Supplement: Figure S1 — Nucleotide and deduced amino acid sequence analysis of NlugCSP8. The predicted putative signal peptides are underlined and denoted by red color. The four conserved cysteine residues are showed in red boxes. The stop codon is indicated with an asterisk. [file Image1.JPEG]

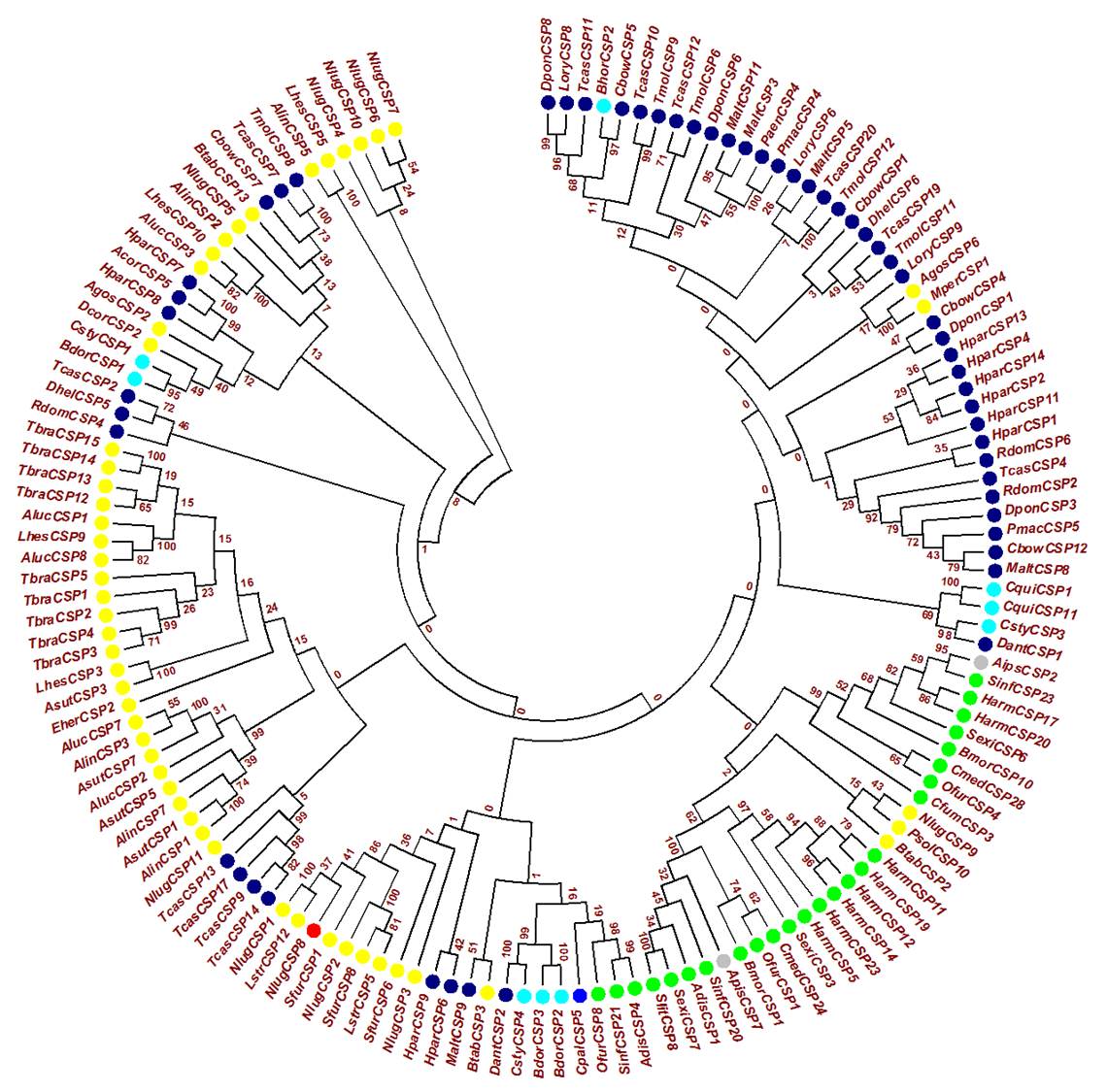

Supplement: Figure S2 — Molecular phylogenetic analysis of amino acid sequences by neighbor-joining (NJ) method. The tree was constructed using the neighbor-joining method with bootstrap support values (%) based on 1,000 replicates. NlugCSP8 are marked with a solid red circle and all other CSPs from Hemipteran are marked with solid Yellow circles. CSPs from Coleoptera, Lepidoptera, Diptera, Hymenoptera, and Neuroptera are marked with Navy, Lime, Aqua, Blue and Silver circles, respectively. All sequences are available from the NCBI database. Species abbreviations are included for taxon identifications. Dpon (Dendroctonus ponderosae), Tcas (Tribolium castaneum), Bhor (Batocera horsfieldi), Cbow (Colaphellus bowringi), Tmol (Tenebrio molitor), Malt (Monochamus alternatus), Paen (Pyrrhalta aenescens), Pmac (Pyrrhalta maculicollis), Dhel (Dastarcus helophoroides), Agos (Aphis gossypii), Mper (Myzus persicae), Rdom (Rhyzopertha dominica), Cqui (Culex quinquefasciatus), Csty (Calliphora stygia), Dant (Delia antiqua), Apis (Apis mellifera), Sinf (Sesamia inferens), Harm (Helicoverpa armigera), Sexi (Spodoptera exigua), Bmor (Bombyx mori), Cmed (Cnaphalocrocis medinalis), Ofur (Ostrinia furnacalis), Cfum (Choristoneura fumiferana), Nlug (Nilaparvata lugens), Psol (Phenacoccus solenopsis), Btab (Bemisia tabaci), Adis (Athetis dissimilis), Slit (Spodoptera litura), Cpal (Chrysopa pallens), Lory (Lissorhoptrus oryzophilus), Bdor (Bactrocera dorsalis), Sfur (Sogatella furcifera), Lstr (Laodelphax striatella), Alin (Adelphocoris lineolatus), Asut (Adelphocoris suturalis), Aluc (Apolygus lucorum), Eher (Euschistus heros), Lhes (Lygus hesperus), Tbra (Triatoma brasiliensis), Acor (Anomala corpulenta), Dcor (Drosicha corpulenta), and Hpar (Holotrichia parallela). [file Image2.JPEG]

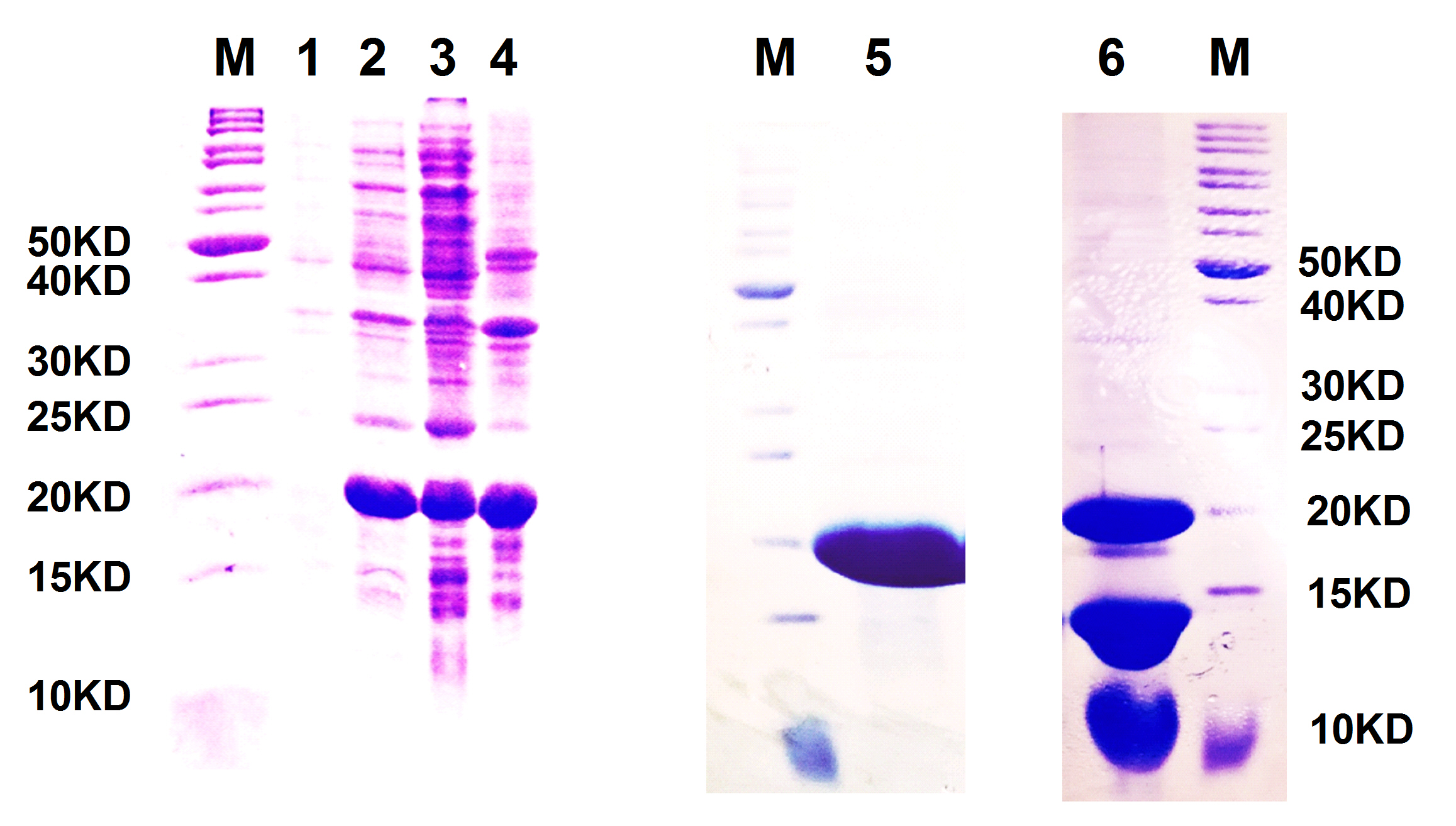

Supplement: Figure S3 — SDS-PAGE analyses showing the expression and cleavage of recombinant NlugCSP8. Lane M: Molecular marker, Lane 1: Non-induced BL21 (DE3) bacteria with pET-30a, Lane 2–4: different IPTG concentrations used to induce recombinant protein (2, 4, 6 mM from lanes 2 to 4), Lane 5: Eluted protein before cleavage, Lane 6: cleaved protein by the recombinant enterokinase. [file Image3.JPEG]

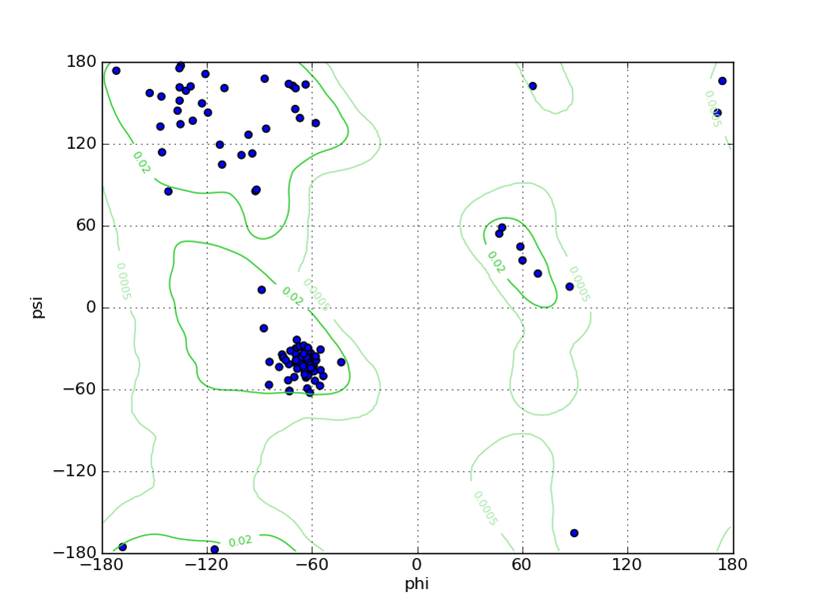

Supplement: Figure S4 — The Ramachandran map for the model of NlugCSP8. [file Image4.JPEG]
